# Newspaper Charlatans

**Published:** 1875-07

**Authors:** 


NEWSPAPER CHARLATANS.—It is the pride of medical men that, in all civilized countries, it is regarded as disreputable for physicians to resort to the newspaper press for notoriety or emolument. This fact is not only well known to the profession, but it has become familiar to a large portion of the secular public. Honorable practitioners carefully abstain from the use of this unfair method of competing for an honest livelihood, or for what is even far more valued, an honest reputation. Indeed, if the really upright and fair physicians of this country be questioned, it will be found that they would as soon defraud or deceive their unprofessional neighbors, as, by the forbidden use of the daily press, to take a mean and dishonest advantage of their professional competitors. Yet, while this is the general belief, the general sentiment of both the professional and unprofessional public, in regard to the use of the daily press by physicians, like many general beliefs and general sentiments, it will be found to be wholly incorrect if tested by the light of truth. Close observers and truthful witnesses have reluctantly to offer very different testimony. There are very many medical men who, claiming to be honorable and upright, are constantly appealing to the daily press for gratuitous notices and unfair advantages. These appeals, it is true, are not always bravely and fairly made, but he must be a sleepy sentinel indeed who does not, through all the ingenious screens of cunning and chicanery used by these enemies of the profession, detect them at their nefarious and infamous work. Such men are members of the medical societies; they claim to be in good standing; to be habitual observers of rules which such societies impose upon their members; to be fair in dealing with their brethren; to be just toward them; to be scrupulously careful of taking no mean and dishonorable advantage of them; to be using society membership for the general good of their profession, and not as an ingenious and contemptible cloak for protecting them in their disreputable practices. They are found in every city, town and village in the land. They are so multiplying as really to form a distinct and treacherous band in all of the camps of the great medical army. If they speak at societies, or conventions, or irregular meetings of physicians, reporters of the daily press are always conveniently present; if they read papers, these are always solicited by the press (!) which, in the sale of large editions, finds emolument for its reprehensible work; papers that are the property of societies before whom they are read, appear in the columns of the daily press; if held by the society's committee on publication and printed in the "Transactions," they appear in the daily press under the touching caption, "from advance sheets of the Transactions;" if an article on semi-professional or sensational subjects be sent for publication to the medical journals, there appears the now familiar reprint in pamphlet form, disseminated broadcast throughout the land; if an epidemic appears, there are newspaper columns of medical nonsense from every unfair professional bidder for notoriety and applause, such articles being invariably a fair reflex of the mind and character of those foolish and unfair enough to furnish them for publication; announcements of the departures and arrivals of small medical men are now familiar paragraphs in every daily paper; lectures to gaping school-boys and innocent school girls are conspicuously announced and often reported; attendance on some prominent patient is regarded as such distinction by the vain and silly, that the reporter learns (always accidentally) the fact; the transient in-dwelling of some celebrity with an ambitious, silly doctor so turns the brain of the poor fellow that he is unhappy until the fact is conspicuously heralded. Indeed, every fact, however unimportant, which can be made the occasion for a paragraph notice of some doctor itching for notoriety is invariably seized upon for this gratuitous and silly form of advertisement.

Is this picture overdrawn, or is it not one too familiar to the American public? Is it not time that this great stain upon the body medical should be expunged, and those responsible for it made to severely and justly suffer for their disreputable course of action? Or shall the profession bear the sins and endure the injury perpetrated by these creatures? Every honest physician feels that he and his profession are deeply injured by them, and that they are making the calling of medicine the most disreputable form of charlatanism and quackery.

How shall this stupendous evil, this public disgrace, be removed? Societies are demonstrably inefficient; they have the power to correct the evil, but not the nerve. Is there no redress? There surely is one; it is for the medical press to make these men odious; to make their practices infamous; to habituate the profession to regard and declare those who thus conduct themselves as frauds and impostors in medicine; as the veriest charlatans, fast dragging a great profession into the mire of public ridicule and contempt.—*Medical Weekly.*

